# The use of artificial intelligence in stereotactic ablative body radiotherapy for hepatocellular carcinoma

**DOI:** 10.3389/fmed.2025.1576494

**Published:** 2025-06-06

**Authors:** Atsuto Katano

**Affiliations:** Department of Radiology, The University of Tokyo Hospital, Tokyo, Japan

**Keywords:** artificial intelligence, stereotactic ablative body radiotherapy (SABR), hepatocellular carcinoma, radiotherapy, treatment planning

## Abstract

The integration of artificial intelligence (AI) into stereotactic ablative body radiotherapy (SABR) for hepatocellular carcinoma (HCC) is transforming the landscape of liver cancer treatment. SABR has emerged as a promising treatment option for patients with localized HCC, offering high local control rates and favorable toxicity profiles. As evidence supporting SABR's clinical efficacy continues to grow, AI technologies are accelerating its adoption by enhancing precision, efficiency, and individualization of care. This review summarizes recent advances in AI applications across the SABR workflow, including automated contouring, knowledge-based planning, fluence prediction via deep learning, respiratory motion modeling, liver function estimation, and prognostic modeling. Clinical studies have demonstrated notable benefits, such as a reduction in contouring time and improved dosimetric quality using machine learning–based optimization algorithms. However, critical limitations persist. Many AI models are trained on limited datasets without external validation, raising concerns about overfitting and generalizability. Future efforts should focus on improving model transparency, confirming their reliability across different institutions, and ensuring ethical use in real-world clinical practice.

## 1 Introduction

Hepatocellular carcinoma (HCC) is the most common primary liver cancer, with a rising incidence worldwide, particularly in Western countries (1). Its development is closely linked to chronic liver diseases, including hepatitis B virus (HBV) and hepatitis C virus (HCV) infections, liver disease caused by excessive alcohol consumption, and metabolic dysfunction-associated steatotic liver disease (MASLD) (2). The distribution of HCC varies by region. East Asia and sub-Saharan Africa have high incidence rates owing to the endemic prevalence of HBV and HCV, whereas in Western countries, the increasing burden of MASLD and alcohol misuse contributes significantly to the rise in HCC cases (3). Unlike alcohol-or viral hepatitis-associated HCC, MASLD-related HCC can develop without cirrhosis, distinguishing it from other forms of the disease and contributing to its rising incidence (4, 5). MASLD-related HCC poses unique problems in surgical treatment owing to its association with metabolic comorbidities, significantly increasing the risk of postoperative complications, including mortality, surgical-site infections, cardiovascular events, and prolonged hospital stays (6).

Stereotactic ablative body radiotherapy (SABR) is used for precise delivery of a high radiation dose to a localized tumor with minimal exposure to the surrounding healthy tissues (7). It has emerged as a promising treatment option for HCC, yielding a high rate of local tumor control, with a 1-year local recurrence rate below 10% (8, 9). Many international guidelines endorse SABR as an alternative or salvage ablative treatment for early-stage HCC (10). According to the American Society for Radiation Oncology practice guidelines, the recommended dosage for non-cirrhotic livers is 40–60 Gy delivered in 3–5 fractions (11). However, several problems remain, including tumor motion due to respiration, the risk of radiation-induced liver toxicity in patients with compromised hepatic function, radiation exposure to adjacent organs, and the complexity in the prediction of the treatment response and long-term outcomes.

**Figure 1:**
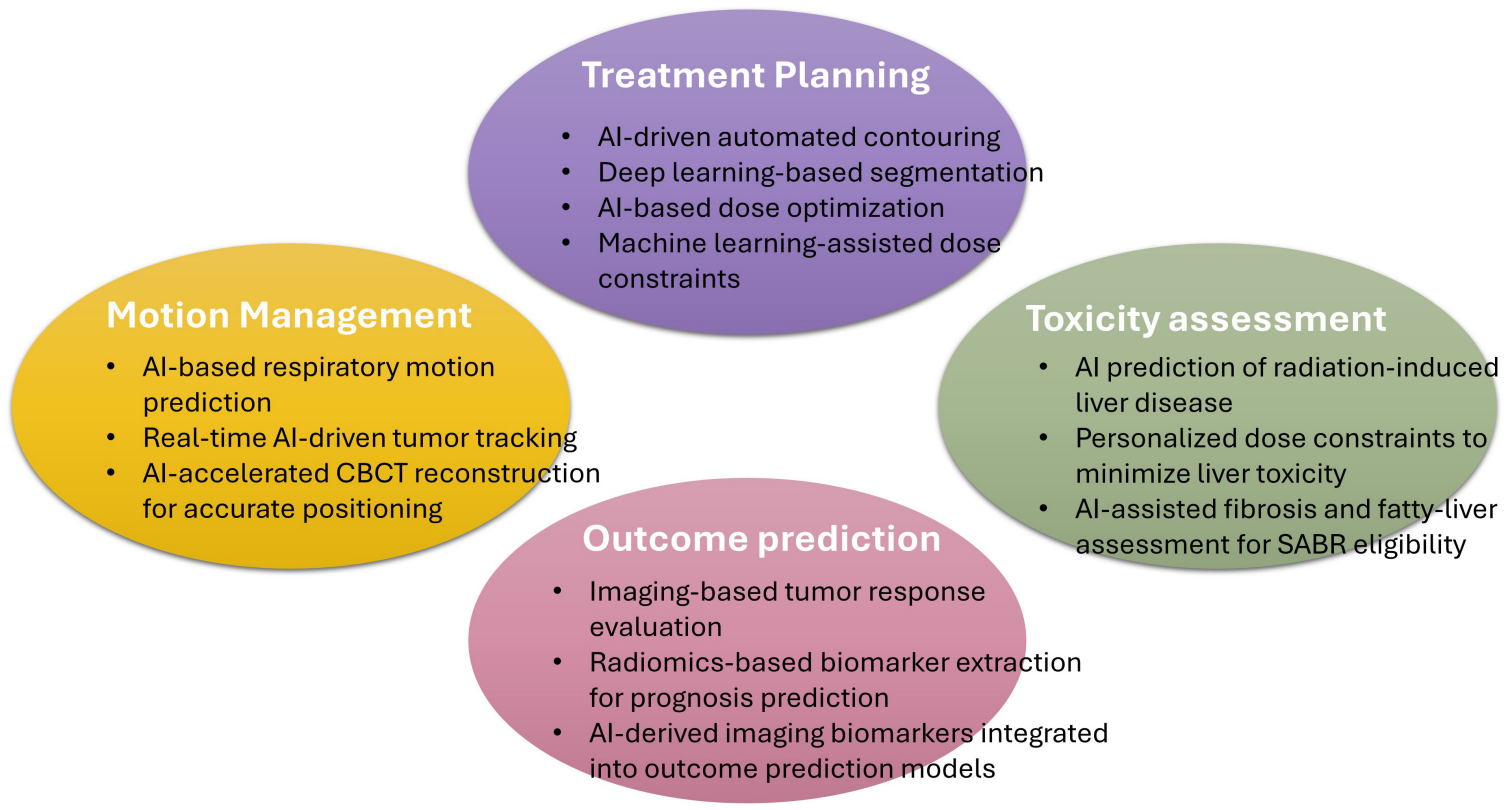
Schematic Representation of Artificial-Intelligence Applications in Stereotactic Ablative Radiotherapy for Hepatocellular Carcinoma. This figure illustrates the major domains where artificial intelligence (AI) contributes to stereotactic ablative body radiotherapy (SABR) for hepatocellular carcinoma (HCC). CBCT, Cone-Beam Computed Tomography.

In recent years, artificial intelligence (AI) has transformed the landscape of oncology, unlocking new opportunities for innovation (12). It offers potential solutions to the abovementioned problems. AI-driven innovations in imaging, motion management, automated treatment planning, and outcome prediction hold promise for treatment precision, safety, and personalization in this field. In this review, we summarize the recent advancements in the application of AI in the field of radiation therapy and examine its potential for implementation in SABR of the liver (Figure 1).

## 2 Optimization of radiotherapy planning

The integration of AI into radiotherapy planning has the potential to enhance efficiency, improve targeting precision, and minimize radiation exposure to normal tissues. By reducing reliance on manual processes, AI mitigates variability in treatment planning, enabling more consistent and high-quality radiotherapy. AI-driven automated treatment planning leverages historical treatment data to optimize beam angles and dose distributions, leading to enhanced dose conformity and workflow efficiency. Automating this process reduces planning time while alleviating the clinical workload, offering substantial benefits in terms of standardization and reproducibility.

Tumor and organ contouring, one of the most labor-intensive aspects of radiotherapy, is subject to inter-observer variability. AI-based auto-segmentation technologies automatically recognize tumor morphology, spatial positioning, and surrounding normal structures, thereby generating high-precision contours in a considerably shorter time than manual segmentation. Wang et al. (13) analyzed a cohort of 36 patients to evaluate the performance of two AI-based auto-contouring software tools. They revealed that both tools generated clinically acceptable contours for ~65% of organs at risk (OAR). However, further refinement is required to enhance the accuracy for more challenging OAR structures and improve the overall model performance. Doolan et al. (14) evaluated five commercially available, AI-based, auto-segmentation solutions. They discovered high geometric similarity between AI-generated and manually contoured structures, with volumetric Dice similarity coefficients ranging from 0.82 to 0.88, and contouring times were reduced by 14–93 min depending on the anatomical site. In the Asia-Pacific context, Kim et al. (15) assessed the performance of seven AI-based auto-contouring systems for OAR segmentation. Their reported that AI-generated contours were clinically acceptable in most cases, although the accuracy was lower for small or anatomically complex structures, necessitating expert review to ensure precision.

The conventional approach to AI-based automated treatment planning is knowledge-based planning (KBP), which utilizes historical treatment data to automatically generate treatment plans for new patients (16). It begins with a training phase, in which expert knowledge and historical cases are used to establish a predictive model that maps patient-specific and plan-related input features to optimal dose parameters. This knowledge base is subsequently applied to new patients, in which their unique characteristics are analyzed to predict tailored dose parameters, ensuring personalized and effective treatment plans. By leveraging machine learning (ML) and historical data, the KBP approach improves planning efficiency, reduces variability, and enhances the precision of radiotherapy to manual planning. Cornell et al. (17) investigated the effectiveness of AI-driven KBP compared to that of manual planning across multiple anatomical sites and discovered that KBP was non-inferior overall. Additionally, dosimetric analyses have confirmed that KBP is effective in reducing plan variability, improving OAR dose constraints, and maintaining treatment efficacy (18). These results underscore the potential of AI to improve treatment standardization and reproducibility across diverse clinical settings.

Another AI-based, automated, treatment-planning approach is deep reinforcement learning (DRL), which has shown promise in its formulation of the process as an optimization problem that balances conflicting OAR protection and target-dose coverage. Li et al. (19) reported that DRL significantly contributed to the automation of radiotherapy plan optimization. However, its widespread clinical adoption remains challenging owing to inefficiencies, limited quality-assessment methods, and a lack of interpretability.

A deep learning-based neural-network algorithm for fluence-map prediction has been developed as a novel approach for radiotherapy planning (20). These predictions are integrated into a treatment-planning system for dose calculation and plan generation, enabling automatic intensity-modulated radiotherapy (IMRT) planning without reliance on the traditional inverse planning process. This system was named automatic IMRT planning via static field fluence prediction (AIP-SFFP) (21). As the inverse-planning process is time-consuming, this approach substantially accelerates the planning workflow while maintaining excellent plan quality. Li et al. (22) demonstrated that the overall isodose distribution of AIP-SFFP-generated plans was comparable to those of KBP and clinical plans. AIP-SFFP generated each test plan, including prediction and dose calculations, within 20 s.

The importance of radiotherapy quality assurance (QA) is underscored by studies showing that deviations from trial protocols are common (23). AI-driven, automated QA systems verify treatment-plan consistency, detect dose-calculation errors, and monitor the calibration of radiation-delivery devices (24). By enabling real-time anomaly detection, human error is reduced and treatment accuracy and patient safety are enhanced. Chan et al. (25) explored ML applications in machine- and patient-specific QA, including system performance monitoring, automated segmentation, and treatment planning. Their virtual IMRT QA system predicted passing rates across institutions, thereby improving treatment accuracy and efficiency. A review of the algorithms, dataset sizes, input features, and clinical applicability of ML and deep-learning models for QA outcome-prediction (26) highlighted their challenges, future directions, and potential impact. AI-based QA will play a crucial role in patient safety.

Recent advancements in SABR for liver cancer include the use of functional imaging to enhance treatment planning (27). Imaging modalities such as dual-energy computed tomography and single-photon emission computed tomography offer valuable insights into tumor metabolism and perfusion, enabling more precise dose-distribution planning (28, 29). The integration of AI with functional imaging has considerable potential for the optimization of real-time, adaptive planning, which would facilitate greater personalization of radiotherapy strategies.

Convolutional neural networks (CNNs) have been widely adopted for medical image segmentation due to their strong performance in spatial feature extraction (30). Recently, transformer-based architectures have emerged as compelling alternatives, demonstrating superior performance in complex tasks such as organ delineation (31, 32). By effectively capturing long-range dependencies and global contextual information, transformers offer distinct advantages over conventional CNNs (33). However, their performance depends heavily on large, annotated datasets and they are computationally demanding, requiring substantial hardware resources. As a result, many current approaches adopt hybrid architectures that integrate CNNs and transformers (34).

## 3 Management of respiratory motion

In liver stereotactic radiotherapy, management of respiratory motion is critical for the accurate delivery of treatment. Various techniques such as breath-hold methods, abdominal compression, respiratory gating, and real-time tracking are employed to address this challenge. Real-time tracking of tumor movement caused by patient respiration substantially improves the accuracy of irradiation. The European Society for Radiotherapy and Oncology guidelines recommend using the diaphragm as a surrogate tracking marker for tumor motion (35) and recent advancements have enabled such tracking by using kilovoltage projection streaming images (36, 37). In addition, infrared reflective markers attached to the patient's skin and fiducial markers are commonly used. Prediction models are used in this approach to learn the relationship between surface-marker motion and internal tumor displacement, thereby optimizing the timing of radiation delivery.

Most prediction models used in clinical practice rely on conventional statistical approaches, such as linear regression (38, 39). However, tumor motion is highly non-linear, complicating the accurate capturing of respiratory motion patterns via traditional methods. Furthermore, variations in the breathing patterns of individual patients complicate the generalization of predictive models, leading to reduced prediction accuracy and potential errors in radiation delivery. To address these limitations, AI technology is being explored to improve the precision of tumor motion prediction (40). AI-driven models are more effectively in capturing the non-linear characteristics of tumor motion, yielding higher accuracy than conventional regression models. The integration of AI into motion tracking holds great promise for radiotherapy precision, safety, and effectiveness for liver tumors.

Zhou et al. (41) developed a convolutional neural network to improve infrared reflective marker-based real-time tracking in radiotherapy. Their AI-driven prediction models demonstrated higher accuracy in the prediction of tumor positions than conventional regression model. Liang et al. (42) developed an AI-based framework to evaluate intrafractional motion via fiducial tracking in patients with liver cancer undergoing robotic SABR. Their framework achieved high accuracy in fiducial marker detection and motion assessment, demonstrating that most treatment fractions exhibited fiducial cohort rotations beyond system limitations; however, rotational correction significantly reduced residual errors.

By enabling high-precision motion prediction, AI has the potential to reduce treatment margins, preserve blood flow, and improve therapeutic efficacy, thereby improving the safety and effectiveness of liver stereotactic radiotherapy.

## 4 Liver-function assessment, toxicity risk prediction, and real-time adaptive radiotherapy

A distinctive feature of radiation-induced liver toxicity is radiation-induced liver disease (RILD), which is characterized by anicteric ascites and hepatomegaly and is primarily caused by microvascular injury (43). Traditional liver-function evaluation relies on clinical scoring systems such as the albumin–bilirubin (ALBI), Child–Pugh, and Model for End-Stage Liver Disease scoring systems, which are derived from pretreatment laboratory data (44). The prediction of toxicity after radiotherapy is based on dosimetric parameters, including the mean liver dose and the functional liver volume spared from radiation (45). However, these conventional approaches have substantial limitations: they cannot effectively capture dynamic changes in liver function, do not account for individual patient variability, and cannot adapt to complex interactions between multiple risk factors. AI-driven methodologies offer potential solutions to these challenges through comprehensive data integration, sophisticated pattern recognition, and adaptive modeling capabilities (46). By analyzing large datasets encompassing clinical, laboratory, imaging, and dosimetric parameters, AI algorithms can potentially provide more accurate and personalized predictions of the liver toxicity risk and enable real-time treatment optimization.

The evaluation of liver function plays a crucial role in clinical decision-making and directly influences the selection of therapeutic interventions. Río Bártulos et al. (47) developed a liver function assessment system that leverages deep learning to analyze magnetic resonance images (MRIs). Their study demonstrated that this AI-driven imaging approach could effectively evaluate liver function compared with the established ALBI score. Wei et al. (48) developed probability models for normal-tissue complications that incorporate voxel-wise functional information from dynamic MRIs to improve patient-specific adaptation to SABR for HCC. The feasibility of their models was demonstrated in a small cohort, with AI-based predictions exhibiting promising accuracy in the estimation of localized liver-function changes. Prayongrat et al. (49) developed an ML-based probability model for normal-tissue complications to predict RILD in patients with HCC, using data of 201 patients. Their study demonstrated an effective ML-based approach to estimate the risk of liver toxicity.

In the field of radiation oncology, adaptive radiotherapy has advanced to allow real-time dose adjustments in response to tumor changes (50). AI has the potential to improve the accuracy and efficiency of adaptive radiotherapy. Deep-learning-based auto-segmentation reportedly reduces the workload of clinicians while achieving high-precision delineation of tumors (51). Additionally, real-time optimization may enable dose-distribution adjustments that account for anatomical changes during treatment (52). Furthermore, AI-driven image analysis may enhance positional correction by using cone-beam computed tomographic images or MRIs, potentially improving irradiation accuracy (53). Moreover, the use of predictive models may help optimize the timing of adaptive treatment, reducing unnecessary plan modifications (54). As these technologies advance, they are expected to facilitate the automation of adaptive radiotherapy workflows and reduce treatment time while maintaining quality.

## 5 AI-assisted prognostication in HCC treatment

Predicting the outcomes of curative local therapies for HCC is crucial for the optimization of follow-up strategies and estimation of patient survival. AI-based predictive models are emerging as valuable tools in this domain, leveraging clinical and imaging data to enhance decision-making and personalize treatment approaches.

Sato et al. (55) developed an ML model to predict the risk of HCC recurrence after radiofrequency ablation (RFA), with the gradient-boosting decision-tree model achieving the highest predictive performance (C-index = 0.67) (55). The model identified the tumor number, serum albumin level, and des-gamma-carboxyprothrombin level as key predictors, enabling personalized risk stratification and follow-up planning. Zandavi et al. (56) created an AI-based model to predict recurrence after surgical resection from the data of 958 patients (56). Their model achieved high accuracy (cross-validation, 0.857; testing, 0.835) by incorporating pre-surgical risk factors, leading to the development of an online tool for real-time prediction of the recurrence risk to facilitate tailored interventions. Hu et al. (57) demonstrated that an AI-driven computed tomography radiomics model could predict progression-free survival (PFS) in patients with colorectal liver metastasis who were undergoing radiotherapy, achieving a C-index of 0.68. Key predictive features of their model include the strength of the gray-tone difference matrix and the maximum radiation dose, highlighting the potential to integrate radiomics with clinical data to enhance prognostication and guide treatment strategies. In a meta-analysis, Wu et al. (58) systematically evaluated the predictive performance of AI for recurrence after first-line liver-cancer treatment. They revealed that AI models achieved high predictive accuracy, with pooled areas under the receiver operating characteristic curve of 0.92 for percutaneous ablation, 0.86 for surgical resection, and 0.79 for transarterial chemoembolization (TACE) in patients with HCC. These results underscore the clinical applicability of AI in recurrence prediction and risk stratification.

Expanding on disease-specific models, Keyl et al. (59) developed AI-derived, cancer-agnostic clinical markers by using multimodal real-world data and explainable AI. By analyzing 15,726 patients with 38 types of solid cancer, they demonstrated that AI-driven assessment of clinical markers may greatly contribute to personalized oncology care via the enhancement of treatment planning and risk prediction across various malignancies.

Multiple options, including surgical resection, RFA, TACE, and SABR, are available for local therapy for HCC. Treatment selection depends not only on tumor stage but also on hepatic reserve, overall patient condition, and individual preferences. The precise selection of individuals for therapeutic strategies with established survival advantages is crucial to improve outcomes. A more refined patient selection approach can be achieved by integrating longitudinal clinical data, predictive modeling, and AI-assisted decision support systems. These advancements have the potential to improve treatment personalization, optimize resource allocation, and ultimately enhance survival outcomes for patients with HCC.

## 6 Limitations in clinical implementation

Several limitations impede the widespread clinical adoption of AI for SABR for HCC. The primary issue is the lack of standardization across AI models and treatment planning systems, which leads to variability owing to differences in training data, contouring protocols, and radiation delivery methods (60). Limited interoperability between systems further impedes integration into diverse clinical environments. And, the absence of unified regulatory frameworks delays approval processes and creates uncertainty regarding compliance and clinical responsibility (61). Additionally, ethical considerations include data privacy, algorithmic bias, lack of transparency, and the challenge of establishing accountability in AI-assisted decision-making (62). Furthermore, Niraula et al. (63) have indicated that both excessive reliance on and excessive skepticism of AI may hinder treatment optimization. Determining the appropriate balance in human–AI collaboration remains a considerable challenge that requires careful consideration. Standardization, rigorous validation, and seamless clinical integration are essential to fully harness the potential of AI.

## 7 Conclusion

The integration of AI into SABR for HCC holds great promise for the enhancement of treatment precision, patient outcomes, and workflow efficiency. AI-driven advancements, including automated treatment planning, real-time tumor tracking, and predictive modeling of treatment responses, have demonstrated considerable potential to improve the effectiveness of radiotherapy.

SABR for HCC is becoming increasingly complex, performed in unison with molecularly targeted therapy and immune checkpoint inhibitors. Dawson et al. (64) conducted a phase 3 trial in which they compared SABR plus sorafenib to sorafenib alone among 177 patients with locally advanced HCC. SABR improved the median overall survival (OS; 15.8 vs. 12.3 months, hazard ratio [HR]: 0.72, *P* = 0.04) and PFS (9.2 vs. 5.5 months, HR: 0.55, *P* < 0.001). Chiang et al. (65) retrospectively compared SABR alone to SABR with immunotherapy (SABR-IO) among 100 patients with unresectable HCC. SABR-IO yielded superior survival outcomes (3-year OS: 63.9% vs. 43.3%, *P* = 0.034), an improved time to progression, and a higher overall response rate (88% vs. 50%, *P* = 0.006). In this increasingly complex therapeutic landscape, AI is poised to play a crucial role in the optimization of treatment approaches. In addition to its applications in radiotherapy, AI has the potential to integrate data from multiple treatment modalities, including surgical interventions, percutaneous local therapies, and systemic treatments such as immunotherapy. Such comprehensive integration may enable AI to propose sophisticated and personalized treatment strategies that consider the full spectrum of available therapeutic options.

AI is transforming radiation oncology by enabling more precise, individualized, and effective treatment approaches. In SABR for HCC, AI holds promise in the optimization of workflows, improving of accuracy, and enhancing of patient outcomes. However, its implementation demands a cautious approach that acknowledges its limitations and ensures the safe and reliable use of AI. Moving forward, continuous research and clinical evaluation of AI are crucial to maximize its potential while ensuring the safe and effective delivery of treatment.
